# Pavement Disease Detection through Improved YOLOv5s Neural Network

**DOI:** 10.1155/2022/1969511

**Published:** 2022-10-13

**Authors:** Yinze Chu, Xinjian Xiang, Yilin Wang, Binqiang Huang

**Affiliations:** School of Automation and Electrical Engineering, Zhejiang University of Science and Technology, Hangzhou 310023, Zhejiang, China

## Abstract

An improved Ghost-YOLOv5s detection algorithm is proposed in this paper to solve the problems of high computational load and undesirable recognition rate in the traditional detection methods of pavement diseases. Ghost modules and C3Ghost are introduced into the YOLOv5s network to reduce the FLOPs (floating-point operations) in the feature channel fusion process. Mosaic data augmentation is also added to improve the feature expression performance. A public road disease dataset is reconstructed to verify the performance of the proposed method. The proposed model is trained and deployed to NVIDIA Jetson Nano for the experiment, and the results show that the average accuracy of the proposed model reaches 88.17%, increased by 4.01%, and the model FPS (frames per second) reaches 12.51, increased by 184% compared with the existing YOLOv5s. Case studies show that the proposed method satisfies the practical application requirements of pavement disease detection.

## 1. Introduction

The detection of pavement disease is an essential part of traffic road maintenance. Improving the accuracy of disease identification and speeding up detection has always been an important research topic in this field. Nature disasters and car accidents damage asphalt pavements. If the damaged roads cannot be found and maintained in time, the service life of the asphalt pavements will be shortened, which will even cause traffic accidents. Early road inspections were mainly manual inspections. People collected pavement disease information through photo sampling and tool measurement [1, 2]. However, with the rapid development of road construction, it is difficult to satisfy the detection requirements of urban roads.

To improve the efficiency of detecting pavement diseases, some researchers proposed the automatic pavement detection method based on computer vision technology. Foucher et al. proposed a road marking recognition method, extracted marking elements before identifying connected components, then extracted them by threshold segmentation and binarization, and marked elements and compared with templates to predict road signs. The dataset used in this experiment is 280 images of 1920*∗*1080 urban environment road scenes. The proposed method can recognize pedestrian crossings and arrow directions [3]. However, the proposed method relies on manually acquired target features and lacks deep feature information, resulting in a low target recognition rate and poor robustness in complex and changeable conditions [4, 5].

Pavement disease detection technology based on automatic 3D imaging has recently emerged. Li et al. developed a method based on deep learning and binocular stereo vision. In this paper, the camera is fixed on horizontal support at the vehicle's rear. The genetic algorithm DenseNet was used to classify pavement diseases, and binocular stereo vision and point cloud processing were used for 3D reconstruction [6]. The results show high accuracy of depth and area. However, the rear camera is prone to collide with other cars during the detection, which is dangerous.

In recent years, pavement disease detection based on deep learning provides a piece of more convenient information for the public security and transportation bureau [7–9]. Although target detection technology has made adequate progress, the performance of pavement disease detection degrades in occluded conditions. When the target has been occluded, the target's appearance information is lost, which makes the CNN (convolutional neural network) more likely to drift and eventually lose the target [10–12]. Xia et al. proposed an improved Kernel correlation filter algorithm to solve this problem. The paper used the optimized kernel correlation filter and color histogram model to make up for the disadvantage of each other. At the same time, the paper proposed adaptive joint weights to adjust the weight proportion of the two models [13]. The results on the OTB-2013 dataset can express that the algorithm can reduce occlusion interference in the object tracking process.

In the tracking algorithm, in order to solve the boundary effect problem caused by using cyclic shift to get training samples [14–16], Zhang et al. designed a multiple feature fusion methods, fused handcrafted features and deep features by adaptive weights, and selected dimensions of channel, temporal, and spatial to perform the grouping features, to enhance the relevance between the features and the correlation filter [17]. This paper conducts extensive experiments on OTB-2013, OTB-2015, TC128, UAV123, and VOT2016. The results show that this algorithm performed better than other prevailing trackers in precision and success rate.

In the object detection task, the occlusion situation and super-resolution reconstruction need to be solved [18, 19]. Image super-resolution reconstruction aims to reconstruct a high-resolution image with rich details from low-resolution images, usually used to detect small objects with low-resolution images. Chen et al. proposed an image super-resolution reconstruction method by using an attention mechanism with a feature map. The proposed method extracts useful features from low-resolution images, combines multiple extracted information with the feature map attention mechanism, and restores more details by adaptively adjusting the channel. The results showed that the PSNR (peak signal to noise ratio) and SSIM (Structural Similarity Index) of the images obtained by the proposed method had been improved [20].

Recently, Huawei's Noah's Ark Lab proposed a new type of end-to-end neural network architecture called GhostNet, which was included in CVPR2020 [21]. This paper proposed a Ghost module to generate more feature maps from cheap operations, which can replace the conventional convolution operation, thereby reducing the FLOPs of the network. Experimental results show that the proposed Ghost module can reduce the computational cost of general convolutional layers while maintaining similar recognition performance.

A lightweight detection network for embedded devices based on YOLOv5s is developed to achieve higher detection speed and accuracy. The main contributions of this paper are as follows:

1. Given the numerous parameters and floating-point operations in the YOLOv5s model, a method of replacing the traditional convolutional layer of YOLOv5s with the Ghost module is proposed, and noise and mosaic data enhancement are added to improve the generalization ability of the model.
2. This paper reconstructs an urban pavement disease detection dataset, including five disease types of manhole covers, cracks, road markings, potholes, and grid cracks under different interference environments, a total of more than 8,400 pieces.
3. compared with other methods to improve the model structure. The comparison experiment includes YOLOv3, the improved YOLOv3, YOLOv5s with the new 104×104 feature layer, and the model of replacing the YOLOv5s backbone network with ShuffleNetv2 and MobileNetv3. Finally, Jetson Nano is used as the deploy carrier, simulating the mobile terminal's target detection task in intelligent driving and displaying the detection results of various models, providing new ideas for the edge computing method of road damage detection.

The rest of this paper is organized as follows.  Section 2 presents the principle of YOLOv5, the Ghost module, the developed YOLOv5 model, and image enhancement.  Section 3 discusses the model training process and the experimental results tested on the embedded device.  Section 4 concludes the presented work.

## 2. Methodology

### 2.1. YOLOv5

**Figure 1:**
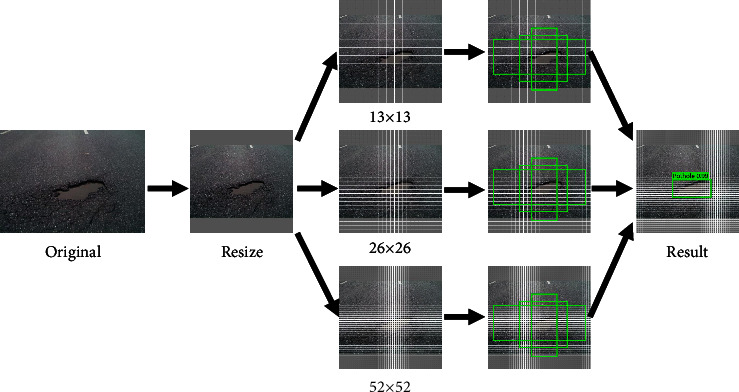
YOLO series detecting process.

The detection speed is very important for road disease detection in intelligent driving, and the one-stage network can output the anchor box and probability of the category in a short time detection process, such as YOLO series are suitable for the task of pavement detection. The main process of the YOLO series model for pavement disease detection is shown in Figure 1.

In this paper, we take the YOLOv5 series as the research object. According to the depth of the network and the width of the feature map, the YOLOv5 series can be classified into four types from large to small: YOLOv5x, YOLOv5l, YOLOv5m, and YOLOv5s. YOLOv5s has the fastest processing speed; therefore, in this paper YOLOv5s is selected for benchmark to minimize the FLOPs and the number of parameters.

**Figure 2:**
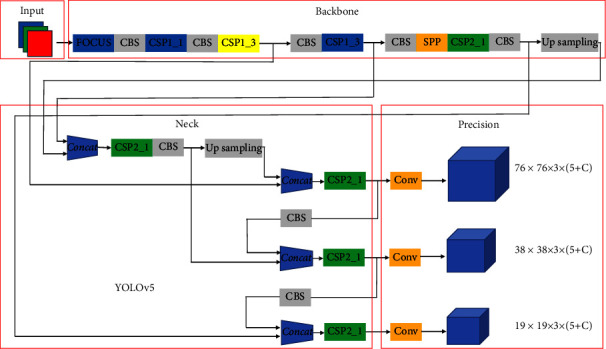
YOLOv5s structure. It consists of four parts: input, backbone, neck, and prediction.

As shown in Figure 2, the YOLOv5s network consists of focus, Conv, C3, SPP(spatial pyramid pooling), and other modules. Conv is the basic convolution unit of the YOLOv5 network. It performs two-dimensional convolution, regularization, and activation operations on the input. C3 module consists of several modules of bottleneck residual structure. The input of the residual structure module goes through two convolution layers and then carries out an add operation with the value to fuse feature information without increasing the network depth.

**Figure 3:**
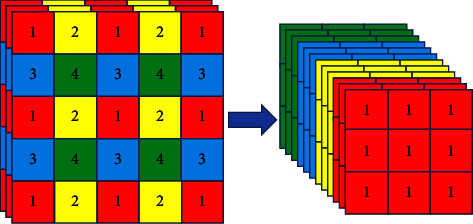
Image slicing operation.

The structure of the focus layer is added to the backbone to enhance the learning ability of the CNN. As shown in Figure 3, the main operation of the focus layer is image slicing. It splits a high-resolution image into multiple low-resolution images, equivalent to sampling every other column, and splices into a higher dimension.

### 2.2. Ghost Module

**Figure 4:**
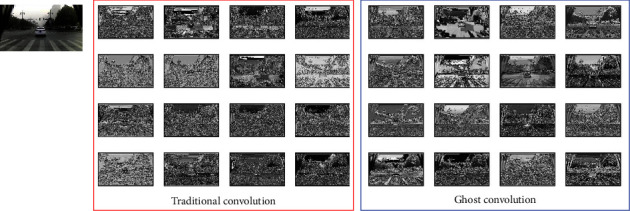
Output feature map comparison.

In the traditional CNN, rich and even redundant feature maps are usually included to ensure a comprehensive understanding of the input data. As is shown in Figure 4, the feature map is taken out by the first convolution layer, the map in the left box is a feature map obtained by the convolution operation, and the right box is a ghost convolution. It can be found that one feature map can be obtained by transforming another feature map with a cheap operation. Therefore, we believe that in the neural network detection of pavement diseases, not all feature maps need to be obtained by convolution operations, and “Ghost feature maps” can be generated to replace some feature maps by cheap operation.

We replaced the original traditional convolution and traditional convolution operations in C3Ghost and GhostBottleneck. The Ghost module is an innovative module proposed in GhostNet. It can effectively obtain more feature maps with fewer parameters and calculation amounts. Unlike other target detection processes, in pavement disease detection, the feature information after convolution is grayscale information, leading to the generation of many repeated feature maps. It also greatly increases FLOPs.

**Figure 5:**
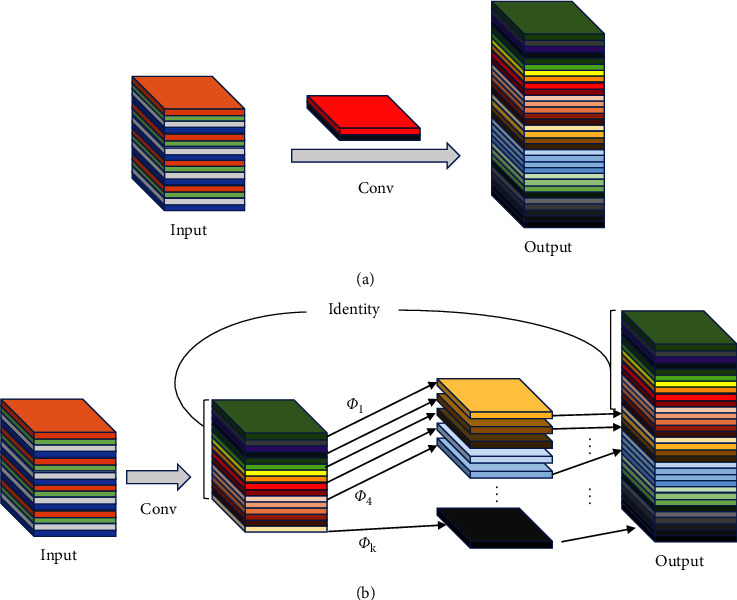
An illustration of the convolutional layer and the Ghost module for outputting the same number of feature maps. Φ represents the liner operation. (a) The convolutional layer. (b) The Ghost module.

The current proposed network models often consist of many convolution operations.  Figure 5(a) shows the input and output results of the convolution layer. Even a 1×1 convolution layer would occupy considerable memory and FLOPs. We proposed that the Ghost module replaces the convolutional layer to solve this problem, as shown in Figure 5(b). The network generates feature maps by less traditional convolution layers and uses generated feature maps to generate new similar feature maps by liner operations. Finally, the information in the two sets of feature maps is combined as output information. In short, Ghost convolution is divided into three steps: traditional convolution, liner operation, and feature map stitching.

### 2.3. Improvement of YOLOv5s Network Architecture Design

**Figure 6:**
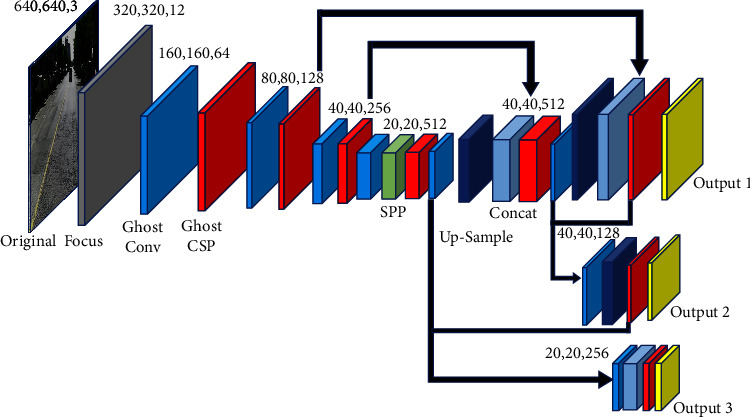
Ghost-YOLOv5s network structure.

A lightweight pavement disease detection network based on YOLOv5s for Jetson Nano is proposed to achieve car detection accuracy and speed. We named it Ghost-YOLOv5s.  Figure 6 shows the schematic diagram of the Ghost-YOLOv5s structure. Each solid rectangle represents a layer. Each number represents this layer's length, width, and the dimension of the feature map output.

Besides, we verified the advantages of the Ghost module with the following data. Assume input data *X* ∈ *R*^*c*×*h*×*w*^, where *c*, *h*, *w* are the number of input channels, the height, and width of the data, respectively, and the operation of an arbitrary convolution layer for generating *n* feature maps can be formulated as follows:

(1) $$\begin{array}{c} Y=X\;*\;f+b\text{,} \end{array}$$

where *∗* is the convolution operation; *b* is the bias;  *Y* ∈ *R*^*n*×*h*′×*w*′^ is the output feature maps; *n*, *h*′, *w*′ are the number of output channels, the height, and width of the output data, respectively; *f* ∈ *R*^*c*×*k*×*k*×*n*^ is the convolution filter in this layer; and *k* × *k* is the kernel size of convolution filter *f*. Therefore, the required number of FLOPs can be calculated as *n*∙*h*′∙*w*′∙*c*∙*k*∙*k* (the bias term is omitted for simplicity), which is often over 10^5^ with the number of filters *n* and the channel number *c* as 256. According to equation (1), the operation of the Ghost module for generating *m* feature maps can be formulated as follows:

(2) $$\begin{array}{c} Y^{\prime }=X\;*\;f^{\prime }\text{,} \end{array}$$

where *f*′ ∈ *R*^*c*×*k*×*k*×*m*^ is the filters (*m* ≤ *n*), and to keep the spatial size(*h*′and *w*′) of the output maps consistent, the filter size, stride, and padding are the same as those in the contradiction convolution. Then, the feature map of each channel of *Y*′ ∈ *R*^*m*×*h*′×*w*′^ uses the *φ*_*i*_ operation to generate several Ghost maps. We assume that the channel number of the feature map is *m*, the number of transformations is *s*, and the number of new feature maps finally obtained is *n*=*m*∙*s*. There are one identity mapping and *m*∙(*s* − 1)=*n*/*s*∙(*s* − 1) linear operations, and the average kernel size per linear operation is *d* × *d*.

Above this, the speed-up ratio of replacing the traditional convolution with the Ghost module is as follows:

(3) $$\begin{array}{c} r=\frac{n∙h^{\prime }∙w^{\prime }∙c∙k∙k}{n/s∙h^{\prime }∙w^{\prime }∙c∙k∙k+\left(s-1\right)∙n/s∙h^{\prime }∙w^{\prime }∙d∙d}=\frac{c∙k∙k}{1/s∙c∙k∙k+s-1/s∙d∙d}\approx \frac{s∙c}{s+c-1}\approx s\text{,} \end{array}$$

where the value of *d* is close to the value of *k*; similarly, the compression ratio can be calculated as follows:

(4) $$\begin{array}{c} r_{c}=\frac{n∙c∙k∙k}{n/s∙c∙k∙k+\left(s-1\right)∙n/s∙d∙d}=\frac{c∙k∙k}{1/s∙c∙k∙k+s-1/s∙d∙d}\approx \frac{s∙c}{s+c-1}\approx s\text{.} \end{array}$$

According to equation (4), it can be concluded that the FLOPs of the ordinary convolution are approximately *s* times of the Ghost module, and the calculation of network parameters is also close to *s*. The depth and width multiple is used to control the depth and width of the YOLOv5 network, which are 0.33 and 0.5, respectively. The Ghost Module, as the convolutional layer of the whole network, can achieve faster detection speed while maintaining depth to meet the requirements required to run on Jetson Nano.

**Figure 7:**
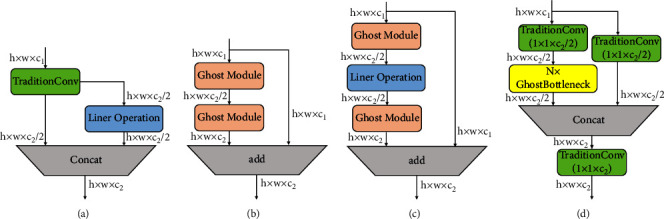
GhostBottleneck and C3Ghost. (a) Ghost module. (b) GhostBottleneck (stride = 1). (c) GhostBottleneck (stride = 2). (d) C3Ghost.

The specific structures of Ghost module, GhostBottleneck, and C3Ghost are designed as shown in Figure 7. The flowchart of Ghost module is shown in Figure 7(a), and the GhostBottleneck consists of several Ghost modules and several liner operations, as shown in Figure 7(d), and the C3Ghost is used to replace the bottleneck module in the C3 module. The new structure can reduce computational costs and compress the network size.

### 2.4. Model Accuracy Improvement

**Figure 8:**
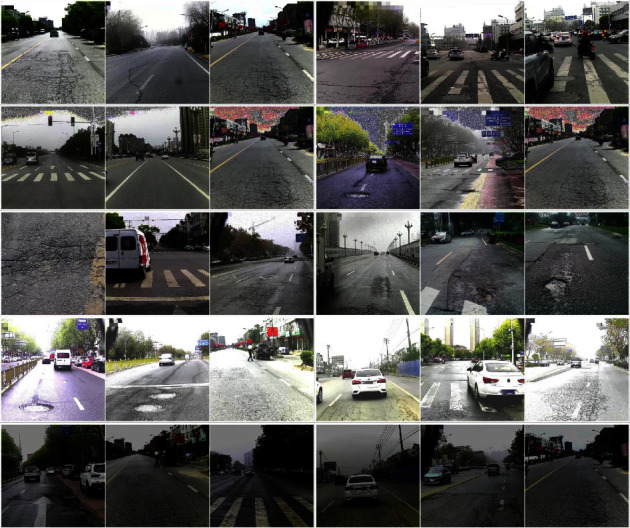
Partial sampling pictures.

In this paper, the Ghost Module can reduce model parameters and FLOPs, and data augmentation is used to improve the detection accuracy of Ghost-YOLOv5s. In this study, we use a self-made dataset, and its basic sample is from urban roads (labeled) in Jiangsu Province. There are 8470 images in total, corresponding to 6830 label files. LabelMe was used to draw polygons for pavement diseases. After that, the XML file, including the road disease's position information, is generated through the python program. The training set contains 6353 images, and the validation set contains 2117 images. It contains five categories: abnormal manholes, cracks, faded markings, net cracks, and potholes.  Figure 8 shows normal sampling images affected by Gaussian noise, salt and pepper noise, excessive illumination, and weak light.

### 2.5. Mosaic Data Augmentation

**Figure 9:**
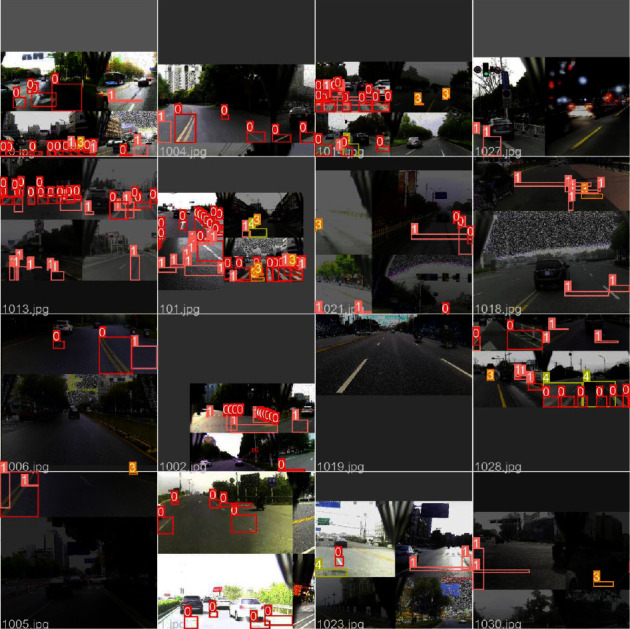
Mosaic data augmentation.

As shown in Figure 9, the mosaic operation randomly selects four images and combines them with random cropping and stitching. The advantage of the mosaic is that all target features of the original images are preserved, and the information of local targets in the dataset is enriched through random stitching and cropping.

## 3. Experimental Procedure

### 3.1. Training Process

In this paper, the training process of the proposed model is run under the PyTorch framework and Linux operating system. The GPU is NVIDIA GeForce RTX2080Ti, and the CPU is Intel Xeon E5-2620v4. The software environment is CUDA 11, CUDNN 7.6, and Python 3.8.

The frozen training method is used for model training to accelerate the convergence of the network. The training iteration is summarized 250 times, and the model is saved once to select the optimal model. 50% of the model weights are frozen for the first 100 times, and the initial learning rate of training is 0.001. After unfreezing the model, reduce the learning rate to 10% so that the network converges faster and reduces the computational burden. The specific process is shown in Table 1.

**Figure 10:**
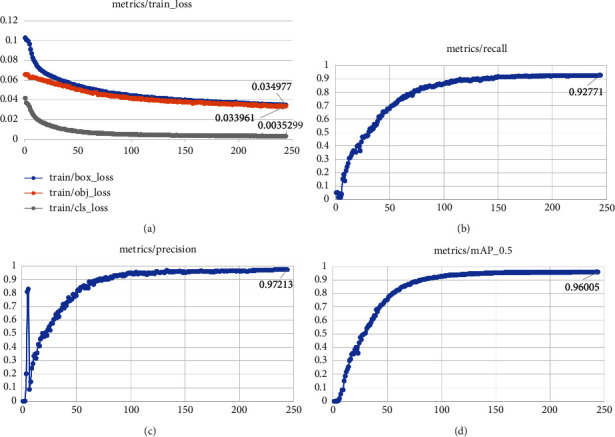
(a) Loss change process curve of Ghost-YOLOv5s. (b) Recall change process curve of Ghost-YOLOv5s. (c) Precision change process curve of Ghost-YOLOv5s. (d) mAP change process curve of Ghost-YOLOv5s.

Figure 10 is the change chart of each indicator during the training of Ghost-YOLOv5s proposed in this paper.  Figure 10(a) is the process that the three loss functions tend to converge gradually after iterations. The dataset has five pavement disease types, and the loss value of the category is close to 0 after 100 iterations.  Figure 10(b) is the training curve of the recall rate, Figure 10(c) is the variation curve of the model precision rate, and Figure 10(d) is the curve of the average precision of the model.

From Figures 10(a)-10(c), the recall rate of the model is stable at 92.771%, the precision is stable at 97.213%, and the mean average precision is stable at 96.005%. From the performance test results of each data in Figure 8, it can be concluded that the proposed Ghost-YOLOv5s network model is ideal in the training phase.

### 3.2. Embedded Device Experiment

**Figure 11:**
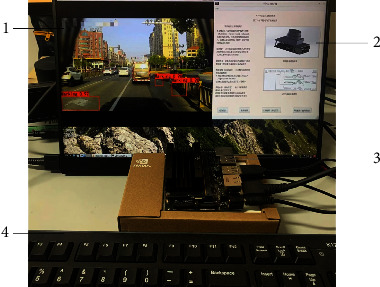
Jetson Nano test chart. 1. Test results; 2. Running interface; 3. Jetson Nano; 4. External keyboard.

After the model training is completed, the model is embedded into Jetson Nano, which includes multiple hardware processing units such as GPU and a supporting tool development environment.  Figure 11 simulates the real-time detection process in the car, 1 is the detection result displayed in real time, and 2 is the human-computer interaction interface, which includes three operating modes:

1. Detecting pictures: detecting the city road pictures at the specified location in the system and saving the detection results in the system folder.
2. Detection video: detect the video of the road when the vehicle is running and save the detection results.
3. Real-time detection: perform real-time detection on the road in front of the vehicle and save the detection results in the specified folder. 3 is the NVIDIA Jetson Nano, which simulates the onboard processor, and 4 is the input device, which is used for debugging the program.

The backbone network of YOLOv5s is replaced with the ShuffleNetv2 and MobileNetv3 network models to show that the proposed model has better detection performance and is more suitable for embedded devices. Compared with the rapidity in time, the target recognition performance is compared with the predicted target's recall rate, recognition rate, and average precision. In addition, to verify the higher detection performance of the newly proposed YOLOv5s model, YOLOv3 and improved YOLOv3 models are added, and all models use the same training set and test set to conduct comparative experiments.YOLOv5s is the smallest version of YOLOv5, which requires less computation and is beneficial to the operation of embedded devices. As can be seen from Table 2, compared with YOLOv3, YOLOv5s has improved performance in terms of parameter quantity, floating-point operations, preprocessing speed, and inference speed. The model Ghost-YOLOv5s proposed in this paper has a preprocessing time of 2.6 milliseconds, an inference time of 72 milliseconds per image, and an FPS of 12.51 frames per second (NMS is the postprocessing method), and the detection speed is faster than other models. The amount of parameters is 2.104 M, and the number of floating-point operations is 2.2 G. Compared with the prototype, the number of parameters is reduced by 4.796 M, the number of floating-point operations is reduced by 14.3 G, and the number of frames processed per second is 2.84 times that of the prototype. After Ghost-YOLOv5s converts the convolution operation into a linear operation, it reduces the parameters by 4.8 M and the memory access amount, making the inference speed on the CPU 28% faster.

**Figure 12:**
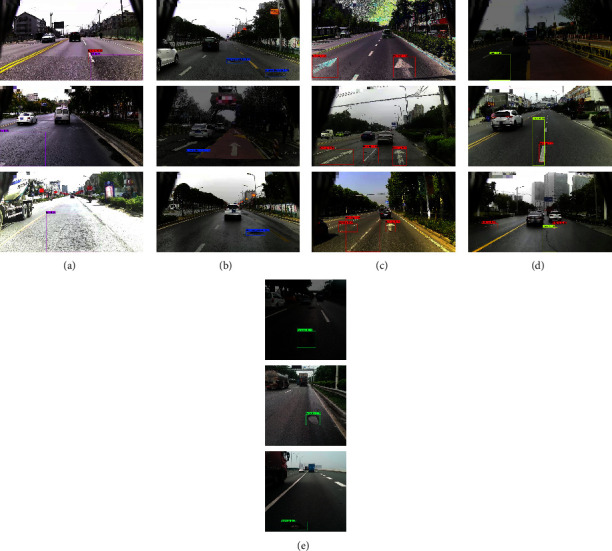
Detect results. (a) Net. (b) Manhole. (c) Marking. (d) Crack. (e) Pothole.

Figure 12 is a part of the test results tested on the Jetson Nano, including potholes, cracks, net, abnormal manholes, and faded markings. In Figure 12, the blue target box indicates that the manhole cover is uneven, the red target box indicates that the mark is faded, the green target box indicates the pothole, the purple target box indicates the grid crack, and the yellow target box indicates the crack. The prediction type and value are displayed on the target box, and the value represents the confidence of the prediction type. The test results show that the algorithm can distinguish five types of diseases, and there is no missed or false detection under multiobjective.

The recognition test results of the model are shown in Table 3. The YOLOv3+Four scale layers add a 104×104 feature layer based on the YOLOv3 structure, and the average precision is increased by 1.13% while the number of floating-point operations increases 9.2 G, which does not meet the speed requirements of this paper's pavement disease detection system. The mosaic data enhancement method can improve mAP by about 0.55% without affecting the detection speed. Replacing the backbone network of YOLOv5s with GhostNet increases the average precision by 1.16%. Experiments are conducted on the proposed methods of SOTA, ShuffleNetv2, and MobileNetv3 to illustrate the performance advantage of the proposed method. The mAP of the Ghost-YOLOv5s proposed in this paper is 10.69% higher than ShuffleNetv2 and 8.82% higher than MobileNetv3.

Compared with replacing the backbone network with ShuffleNetv2 and MobileNetv3, its average precision increases by 0.63% and 0.05%, respectively. Similarly, when replacing the backbone network and all convolutional layers in the network with Ghost convolutional layers, the average precision is 2.92% higher than that of the YOLOv5s prototype. Compared with the improved YOLOv5s model, whose backbone network is ShuffleNetv2 and MobileNetv3, the FPS of the model proposed in this paper is higher, and the mAP is also improved by 2.39% and 1.81%.

Since the urban road image itself is dominated by gray pixel values, it is more likely to generate redundant information and lack target feature information after the output of the convolution layer. The linear operation of the Ghost convolution layer can not only avoid the elimination of effective information, but it can also increase the feature information and improve the model's generalization.

Compared with the YOLOv5s algorithm, the improvement proposed in this paper not only reduces the model size and computation but also improves the average precision, which improves the cost performance of the model and is suitable for processors with insufficient computing power.

## 4. Conclusion

This paper proposes an improved YOLOv5s pavement disease detection algorithm, and a real-world urban pavement disease dataset is created, which addresses the challenges of applying YOLOv5 in object detection. The main conclusions are as follows:

1. The traditional convolution layer is replaced with the Ghost module, which reduces the number of floating-point operations and memory access of the model and speeds up the model inference.
2. The detection performance of the YOLOv5 is improved by mosaic data enhancement and adding noise.
3. Experiments are carried out on Jetson Nano to simulate the use of embedded devices for pavement disease detection on urban roads. The proposed algorithm compresses the number of parameters and improves the inference efficiency while maintaining the detection performance compared with the traditional lightweight network.

The experimental results show that the proposed algorithm has lower computation and higher detection accuracy and satisfies the practical application requirements of pavement disease detection. The proposed method has strong applicability and achieves excellent detection performance on Jetson Nano, reducing the platform storage and computation requirements and having higher detection performance than existing models. Future work will focus on improving the accuracy of crack detection and refining the presented algorithm and methodologies in real applications.

## Figures and Tables

**Table 1:** Training process.

Algorithm 1. training of Ghost-YOLOv5s
**Input:** Training set *D*=(*x*^(*n*)^, *y*^(*n*)^)^*N*^, validation set *V*, learning rate *α*, momentum *m*, regularization factor *λ*, number of network layers *L*, number of neurons *M*_*l*_, 1 ≤ *l* ≤ *L*, and model training parameters *N*_1_, *N*_2_ … *N*_*n*_ (e.g., *N*_1_=10, *N*_2_=30).
(1) Initialize the network parameters using Gaussian.
(2) Randomly sort the samples of the training set;
**//Train the Ghost-YOLOv5s Network**
(3) **for**epoch ∈ *e ***do**
(4) **for***n* ∈ batch**do**
(5) Pick samples (*x*^(*n*)^, *y*^(*n*)^) from the training set *D*;
(6) Feedforward computes the layer input *z*^(*l*)^ and activation value *a*^(*l*)^ for each layer until the next layer.
(7) Backpropagation calculates the error *δ*^(*l*)^ for each layer.
**//Calculate the derivative of each layer**
(8) $\forall l,\partial L\left(y^{\left(n\right)},{\hat{y}}^{\left(n\right)}\right)/\partial w^{\left(l\right)}=\delta^{\left(l\right)}{\left(a^{\left(l-1\right)}\right)}^{T}$;
(9) $\forall l,\partial L\left(y^{\left(n\right)},{\hat{y}}^{\left(n\right)}\right)/\partial b^{\left(l\right)}=\delta^{\left(l\right)}$;
**//Update parameters**
(10) *W*^(*l*)^ ← *w*^(*l*)^ − *α*(*δ*^(*l*)^(*a*^(*l* − 1)^)^T^+*λW*^(*l*)^);
(11) *b*^(*l*)^ ← *b*^(*l*)^ − *αδ*^(*l*)^;
(12) **end for**
(13) **end for**
**Output:** the final converged whole model.

**Table 2:** Comparison of different model performance metrics.

Model	*Pre-T* (ms)	*Inference* (ms)	*NMS* (ms)	*FPS*	*Parameter* (M)	*FLOPs* (G)
YOLOv3	4.0	311	388	3.2	40.585	24.5
YOLOv3 + Four scale	4.0	331	388	3.0	55.792	33.7
YOLOv5s	2.7	223	301	4.4	6.900	16.5
YOLOv5s + Ghostbackbone	2.7	128	301	7.7	4.866	9.5
YOLOv5s + ShuffleNetv2	2.7	109	248	9.0	0.811	1.6
YOLOv5s + MobileNetv3	2.7	92	231	10.6	1.417	0.8
Our Ghost-YOLOv5s	2.6	72	301	12.5	2.104	2.2

**Table 3:** Comparison of different model performance metrics.

Model	*Recall/%*	*Accuracy/%*	*mAP/%*
YOLOv3	51.43	82.16	73.93
YOLOv3 + Four scale layers	52.86	83.74	75.06 (73.93 + 1.13)
YOLOv5s	72.31	92.31	84.77
ShuffleNetv2	69.92	82.57	77.48
MobileNetv3	71.62	81.45	79.35
YOLOv5s + Mosaic	72.42	92.94	85.32 (84.77 + 0.55)
YOLOv5s + Ghost_backbone	73.35	92.79	85.93 (84.77 + 1.16)
YOLOv5s + Ghost_backbone + Mosaic	73.38	93.17	86.44 (84.77 + 1.67)
YOLOv5s + ShuffleNetv2	73.27	92.58	85.30 (84.77 + 0.53)
YOLOv5s + ShuffleNetv2 + Mosaic	73.44	93.21	85.71 (84.77 + 0.94)
YOLOv5s + Mobilenetv3	74.08	93.01	85.88 (84.77 + 1.11)
YOLOv5s + Mobilenetv3 + Mosaic	74.56	93.41	86.29 (84.77 + 1.52)
Our Ghost-YOLOv5s	76.39	95.18	87.69 (84.77 + 2.92)
Our Ghost-YOLOv5s + Mosaic	76.97	95.56	88.17 (84.77 + 3.40)

## Data Availability

The data that support the findings of this study are available from the corresponding author, Yinze Chu, upon reasonable request.
